# The best reviewers of International Brazilian Journal of Urology in 2022

**DOI:** 10.1590/S1677-5538.IBJU.2023.01.02

**Published:** 2023-01-26

**Affiliations:** 1 Universidade do Estado do Rio de Janeiro - Uerj Unidade de Pesquisa Urogenital Rio de Janeiro RJ Brasil Unidade de Pesquisa Urogenital - Universidade do Estado do Rio de Janeiro - Uerj, Rio de Janeiro, RJ, Brasil; 2 Hospital Federal da Lagoa Rio de Janeiro RJ Brasil Serviço de Urologia, Hospital Federal da Lagoa, Rio de Janeiro, RJ, Brasil

In 2022 the International Brazilian Journal of Urology received the highest impact factor of his history and this fact was possible because the serious peer review process of our Journal (1). In this year we received more than 600 papers. The Editor-in-Chief would like to thanks all the reviewers and specially to the Doctors: Alexandre Danilovic (Hospital das Clínicas da Faculdade de Medicina da USP -São Paulo, SP, Brasil); Eduardo Mazzucchi (Universidade de São Paulo - USP, São Paulo, SP, Brasil); José C. Truzzi (Universidade Federal de São Paulo – UNIFESP, SP, Brasil); Márcio Averbeck (Departamento de Urologia, Hospital Moinhos de Vento, Porto Alegre, RS, Brasil); José I. Nolazco (Department of Urology, Brigham and Women’s Hospital, Boston, Massachusetts, USA Instituição?); Caroline Silva (Departamento de Saúde, Universidade Estadual de Feira de Santana - UEFS, Feira de Santana BA, Brasil); Giuseppe Simone (Department of Urology, IRCCS Regina Elena National Cancer Institute, Rome, Italy); Ernesto Reggio (Uroclínica de Joinville Ernesto Reggio, Joinville, SC, Brasil); Renato N. Pedro (Universidade Estadual de Campinas - UNICAMP, Campinas, SP, Brasil) and Yigit Akin (Department of Urology, Izmir Katip Celebi Universitesi Tip Fakultesi, Izmir, Turkey), who reviewed more than 3 articles during the year and strictly within the deadline.

Thanks a lot!!!!!

## References

[1] Margalida A, Colomer MÀ (2016). Improving the peer-review process and editorial quality: key errors escaping the review and editorial process in top scientific journals. Peer J.

